# On the Use of the Tinct. Ferr. Chlorid. in Uterine Hemorrhages

**Published:** 1856-02

**Authors:** Frederick Schreier

**Affiliations:** Physician in Hamburgh


### SELECTIONS.

*On the Use of the Tinct. Ferr. Chlorid. in Uterine Hemorrhages.*—By Dr. FREDERICK SCHREIER, Physician in Hamburgh.

(Translated for the Medical Examiner.)

Experience having shown that all the means generally used for the suppression of uterine hemorrhages—especially when violent—were inefficient, I was induced, after trying various remedies, to employ the liq. stypticus loofi. Not only was my first experiment, made thirteen years ago, attended with success, but I have lately had frequent opportunities of collecting from my colleagues, proofs of still more favorable results, from the use of this medicine in their practice. The history of two cases will best illustrate the practical working of the liq. ferr. chlorid.

In the beginning of the year 1842, I was called to visit the midwife B., in Altenwerder, Hanover. This woman, 26 years of age, of delicate constitution, complained of violent pain in the right lumbar region. On examination, I observed a swelling, which bore all the characteristics of the first stage of an abscess. After applying the usual means for producing suppuration, it opened, and was healed. About 14 days afterwards, Mrs. B., being sent for to attend a woman about to be confined, was obliged to cross the ice on horseback. The horse broke through the ice, and the woman fell into the water. On the next day a violent hemorrhage took place, to stop which she used all the well known means, but in vain. Called to see her; I ordered tinct. cinnamon 1—3 teaspoonful every quarter of an hour, with no effect, however, as the bleeding continued undiminished. A threatened miscarriage could not now be mistaken. Weak, irregular labor pains had commenced, and I was satisfied that the fœtus would have to be expelled from the uterus. I now prescribed: ℞. Secal. cornut. gr. x., rad. ipecac. gr. j., alumin. crud. gr. jv., sacch. alb. gr. vj., M. f. pulv., to be given every hour. I ordered the most perfect rest, horizontal position, and laid a cold sand bag over the pubic region. After this the hemorrhage became less.

On the evening of the same day, I was informed that not only had the bleeding not ceased, but that it had become much more violent, and the woman very weak. I at once ordered: ℞. Decoct. rad. krameriæ ℥vj., tinct. cinnam. ℥½., syr. cinnamon ℥j., acid phosph. ʒij. M. D. S., one teaspoonful every hour, and hurried, suspecting great danger, to the sick woman, whom I found suffering from severe hemorrhage. After examining the mouth of the womb (which was so contracted as to render the insertion of the finger impossible) I applied restoratives (besides injections of cold water, alum and vinegar) after which the bleeding ceased; but, for this time also, only for a short period; for in the night, towards 12 o'clock, a message was sent to me that the bleeding had again commenced, the patient being entirely exhausted, and dying. Upon this I resorted to the energetic measures which I had before used in similar cases, with such favorable results, namely, injections of diluted liquor stypt. loofi. (about 50 drops of liq. stypt. l. in 3—4 oz. of water). Finding after this injection that no sensation of heat was felt in the womb, that the bleeding was unchecked, and that no perceptible contraction of the uterus had taken place, I now gave, instead of 50, 80 and finally 100 drops in 3 oz. of water. The wished for result at once took place. The passage of black coagulated blood with the fœtus followed the injection. The fœtus appeared about 12—13 weeks old, which accorded with the woman's reckoning. Afterwards occurred gastrosis with tympanitis, &c., &c., which was arrested by the appropriate means, and the patient soon completely recovered. She afterwards described her sensations when the above means were used in the following manner: "Upon the first injection I felt a slight warmth, but upon its being repeated, after being made stronger, a most decided heat was experienced in the womb, which increased to a burning sensation, accompanied with a peculiar feeling of motion there, after which I was not aware of any further loss of blood."

I will now relate another case equally interesting.

Mrs. F., at Finkenwarder, 40 years old, of strong constitution, choleric temperament, was seized with hemorrhage. She summoned an esteemed colleague, Dr. N., in N., to her assistance. After trying all the known and valued means in vain, the patient called me in. I resorted again to the injection of liq. stypt. loofi., for the patient had now become almost anæmic, and I had again the satisfaction of witnessing the successful results of its action. After the third injection—in which were 100 drops of liq. stypt. l.—the bleeding ceased. In consequence of the great loss of blood, general dropsy afterwards took place. The cause of the dropsy was clear, and I treated it therefore with iron and other restoratives, and soon checked it. After the course of three years the woman was again pregnant, and was delivered of a living, mature child.

In cases where paralysis of the uterus occurs, and the power of contraction is wanting, I can also recommend to my brethren, as a safe and effective measure, the use of the liq. stypt. l. In the last year of my practice I used the liquor ferr. chlorid. also for a violent hemorrhage in a case of placenta prævia, where rapid delivery could not be effected, owing to insufficient dilatation of the mouth of the womb. I applied it in the following manner: I took a compressed sponge (sponge tent) which was cut in the form of the opening of the mouth of the womb, saturated it with the liquor, and introduced it as high as possible into the mouth of the womb, upon which not only was the bleeding checked, but after the course of 6—12 hours, in consequence of the detention, the dilatation was so increased as to allow me to introduce the whole hand and complete the delivery without any injury whatever. And, indeed, in my practice I have never, in consequence of an artificial delivery, lost a single patient. In uterine cancers, where profuse bleeding frequently occurs, the same remedy can also be recommended.—*Monatsschrift fur Geburtskunde und Frauenkranpheiten, June*, 1855. *Medical Examiner.*

